# Physical Therapist-Led Initiatives for the Prevention and Improvement of Chronic Pain Among Workers: A Case Study of Hosting Workshops Based on Survey Results in a Corporate Setting

**DOI:** 10.3390/ijerph21121709

**Published:** 2024-12-23

**Authors:** Yasumasa Oka, Michio Wachi, Noriyuki Kida

**Affiliations:** 1 Kanazawa Orthopaedic and Sports Medicine Clinic, Shiga 520-3016, Japan; 2 Graduate School of Science and Technology, Kyoto Institute of Technology, Kyoto 606-8585, Japan; 3 Department of Physical Therapy, School of Health Sciences, Bukkyo University, Kyoto 604-8418, Japan; m-wachi@bukkyo-u.ac.jp; 4 Faculty of Arts and Sciences, Kyoto Institute of Technology, Kyoto 606-8585, Japan; kida@kit.ac.jp

**Keywords:** industrial physical therapy, prevention, chronic pain, musculoskeletal disorder

## Abstract

Industrial physical therapy (IPT) interventions by physical therapists can enhance labor productivity. However, in Japan, there is a scarcity of case studies involving corporate visits, questionnaire-based data, and insights into corporate demands. Addressing this gap is vital for improving presenteeism related to chronic pain and increasing employees’ health literacy, thereby advancing corporate health management. This case study evaluates the effectiveness of a workshop aimed at preventing and reducing chronic pain among employees in the Development Department of Company A, an information technology (IT) firm. The research employed pre- and post-survey questionnaires, workshop interventions, and meetings with corporate management to assess the current state of musculoskeletal chronic pain and productivity losses and to verify the intervention’s effectiveness. Approximately 50 participants attended the workshop in person, while around 30 participated online, totaling 80 attendees. A total of 56 (51 men and 5 women) individuals responded to the pre-workshop questionnaire, and 28 responded to the post-workshop questionnaire. The age distribution of the 56 pre-survey respondents was as follows: 9 in their twenties, 13 in their thirties, 22 in their forties, and 12 aged 50 and older. Preliminary survey results showed that 55.4% of participants experienced chronic pain in at least one body part. The average presenteeism value was 82.8% (standard deviation = 16.8). It was also found that literacy regarding appropriate pain management strategies was low. An independent *t*-test comparing literacy scores based on the presence or absence of pain showed no significant differences (*p* = 0.34). Additionally, a one-way ANOVA conducted to examine differences across four age groups revealed no significant differences (F = 0.934, *p* = 0.431). Results from the post-workshop questionnaires indicated that more than 70% of the employees experienced an increase in understanding and satisfaction, with positive feedback on the improvement of knowledge about chronic pain mechanisms and posture. However, there were also requests for more interactive communication and a desire to learn more about specific care methods, suggesting the need to provide interventions tailored to appropriate stages of preventive medicine.

## 1. Introduction

In Japan, the progression of an aging society with a declining birthrate has been widely recognized as causing dual challenges: a decrease in the labor force and an increase in healthcare costs associated with social security. The working-age population in Japan peaked at just under 70% between 1991 and 1993 but has since declined, reaching 59% by 2016, which is the lowest among the Group of Seven countries [1]. Addressing these issues requires an expansion of the labor force and an improvement in labor productivity. From this perspective, the Ministry of Economy, Trade and Industry has emphasized “Health Management” since 2014, incorporating health management into business strategies, with the goal of enhancing productivity [2]. The scope of health management lies within the field of occupational health, which, based on the Occupational Safety and Health Act, implements measures to protect workers’ health. In particular, physical therapy within this field, known as industrial physical therapy (IPT), has been recognized and established as effective in industrialized countries, such as the United States, Australia, and the Netherlands. IPT contributes to the improvement of national health by preventing work-related injuries, improving ergonomic work postures, and enhancing physical function in older workers [3]. Recent advancements have also demonstrated the validity of using noninvasive methods such as infrared thermography combined with raster stereography to assess posture in healthy individuals. These developments are increasingly expanding the potential applications of physical therapy in the field of industrial health [4].

Health-related factors targeted by IPT include those that inhibit continued employment, such as low subjective health perception, chronic diseases, obesity, and lack of exercise [5]. Chronic diseases have a notable impact on labor productivity through presenteeism. Labor loss is classified into absenteeism, which indicates sick leave due to general health problems, and presenteeism, where workers attend work but have a reduced capacity to perform due to health issues [6]. The decrease in work performance attributed to presenteeism has been reported to be approximately three times the cost of absenteeism [7]. In observing the loss per person due to presenteeism among Japanese workers, mental disorders, neck pain/shoulder stiffness, and lower back pain have been found to be the most significant [8]. These facts underscore the considerable impact of chronic musculoskeletal diseases on the labor productivity of workers in Japan and emphasize the necessity for intervention through IPT [9,10].

Chronic pain is significantly associated with chronic musculoskeletal diseases. According to the International Association for the Study of Pain, chronic pain is defined as “pain that persists beyond the normal time expected for healing, or pain associated with progressive non-cancer diseases”. Typically, pain that persists for more than three months is considered chronic. In the revised International Classification of Diseases (ICD-11) of 2018, chronic pain is classified into two major categories: chronic primary and secondary pain [11]. Within these categories, chronic musculoskeletal pain, irrespective of whether primary or secondary, has the potential to affect presenteeism as previously mentioned, thereby directly linking its prevention and improvement to the enhancement of labor productivity [12]. Therefore, to advance IPT, it is necessary to develop effective interventions to address these factors.

Improving chronic musculoskeletal pain also necessitates the enhancement of workers’ health literacy. Health literacy is “the cognitive and social skills that determine the motivation and ability of individuals to access, understand, and use information in ways that promote and maintain good health” [13]. Numerous studies have shown that the level of health literacy significantly affects pain management and quality of life in patients with chronic pain [14,15]. It has been reported that individuals with low health literacy experience significantly greater chronic pain intensity [16]. Thus, improving health literacy could contribute to better pain management of chronic pain.

Based on the above, it is important for corporations to enhance health management by reducing presenteeism caused by chronic pain and increasing health literacy related to chronic pain. Active micro-breaks during work hours, which include stretching and strength-building exercises conducted by physical therapists, have been reported to reduce employees’ pain and fatigue and enhance their mood at work [17]. This supports the growing recognition that industrial physical therapy has the potential to contribute to increased labor productivity. However, despite the Japanese Physical Therapy Association having 139,556 registered members [18], as of November 2022, the Japanese Industrial Physical Therapy Research Society has only 530 registered members [19]. This discrepancy suggests that IPT has not yet become widespread in Japan. Additionally, there is a lack of case reports from onsite corporate visits, data based on questionnaire surveys, and an accumulation of corporate demand data. To advance IPT in Japan, it is essential to demonstrate the effectiveness of these interventions.

Given this background, the general objective of this study was to conduct workshops led by physical therapists aimed at preventing and improving chronic pain among corporate employees. Several specific objectives were also set, including conducting surveys and meetings before and after the workshops to understand the current impact of chronic pain on labor productivity and to evaluate the effectiveness of the interventions implemented in this case. We hypothesized that there are areas for improvement in the current state of musculoskeletal chronic pain among corporate employees and that this intervention would receive a positive evaluation. Based on this hypothesis, we conducted our study.

## 2. Materials and Methods

### 2.1. Study Design

This study is a case study that evaluates the effectiveness of a workshop aimed at preventing and improving chronic pain among employees of the Development Department at Company A, an information technology (IT) company. The research combined pre- and post-survey questionnaires, workshop interventions, and meetings with corporate management to assess the current state of musculoskeletal chronic pain and productivity losses in the department and to verify the effectiveness of the intervention.

### 2.2. Setting

The workshop was conducted at Company A in late December 2023. Prior to the workshop, a preliminary meeting was held in early December 2023 with four individuals: the department head, a representative from the Human Resources (HR) Department, a representative from the General Affairs Department, and the physical therapist responsible for the workshop. The pre-workshop questionnaire was conducted one week prior to the workshop via Google Forms. The post-workshop questionnaire was administered on paper immediately following the conclusion of the workshop. In early January 2024, a post-meeting was held with four individuals who had participated in the preliminary meeting to reflect on the workshop and discuss future research directions. Company A has been selected for the ’Excellent Corporations for Health Management 2023’ White 500 by the Ministry of Economy, Trade and Industry [2]. The company, an IT firm, primarily engages in system and software development and employs approximately 3000 people across the group. The particular facility targeted in this study has about 300 employees.

### 2.3. Participants

The participants were employees from the Development Department of Company A, which consists of approximately 90 system engineers primarily responsible for product development. Their tasks are predominantly desk-based but occasionally involve lifting objects weighing up to 20 kg.

Inclusion criteria were full-time employees of Company A’s Development Department, ranging from their twenties to their sixties, who voluntarily consented to participate in the workshop and could understand instructions given in Japanese. Exclusion criteria included employees currently on maternity or paternity leave, extended sick leave, and those with serious health conditions (e.g., fractures, severe musculoskeletal disorders, cancer) that precluded normal work activities or workshop participation.

The sample size was determined using G Power software (version 3.1.9.7; Heinrich Heine University of Düsseldorf, Düsseldorf, Germany). A power analysis was conducted with a medium effect size, a significance level of 0.05, and a statistical power of 0.80. The analysis indicated that approximately 64 participants per group, for a total of 128 participants, were required.

Recruitment was conducted via email by the HR department to employees who met the inclusion criteria and did not fall under the exclusion criteria. Participants had the option to attend the workshop either in person or online, according to their convenience. Ultimately, about 50 attended in person and 30 online, totaling 80 participants. Fifty-six responded to the pre-workshop questionnaire, and twenty-eight to the post-workshop questionnaire.

### 2.4. Data Collection Tools

#### 2.4.1. Pre-Workshop Questionnaire

The workshop questionnaire was administered online using Google Forms one week prior to the workshop date. The preliminary questionnaire survey included items on basic information such as gender and age group. Subsequently, questions related to chronic pain were asked, requiring participants to answer, using a dichotomous scale, whether they experienced chronic pain lasting more than three months and occurring at least twice a week in any of the three specific body parts (neck, shoulder, and lower back) within the last year. The definition of chronic pain for this study was established based on previous research and chronic pain guidelines [20,21]. While prior studies often defined chronic pain as pain with a Visual Analog Scale (VAS) score of 5 or above, this survey included milder pain levels as well, considering their potential impact on labor productivity. A supplementary explanation was provided to exclude acute pain from injuries such as fractures or cancer-related pain. Regarding exercise habits, participants were asked about their regular physical activity (lasting more than 30 min per day) that they had maintained for over a year (response options included none, once a week, twice a week, or more than three times a week). Additionally, they were asked whether they incorporated short exercises or stretches of approximately 5–10 min into their daily routine, again using a dichotomous scale. For presenteeism, a single-item measure from the University of Tokyo was utilized, asking participants to rate their work performance over the past four weeks compared to their best possible performance, with the presenteeism value calculated as 100% minus the response value. The validity of the method for calculating the presenteeism score has been demonstrated [22]. Regarding chronic pain literacy, seven questions were presented concerning knowledge about chronic pain management (e.g., the role of poor posture at work, importance of resting, irrelevance of productivity decline, influence of physical stiffness, connection to mental health issues, necessity of actively moving the painful area, and effectiveness of cooling the affected area with patches), with participants responding “agree” or “disagree” using a dichotomous scale.

#### 2.4.2. Post-Workshop Questionnaire

The post-workshop questionnaire was administered on paper to those who attended in person on the day the workshop concluded. For the post-workshop questionnaire, participants were asked to rate on a five-point Likert scale whether they were able to gain knowledge from the workshop, understand the content, were inclined to practice stretches or other activities taught, or were satisfied with the workshop. Additionally, regarding the services desired from IPT, the participants could choose multiple options, including workshop attendance, individual consultations, individual care (such as massage or stretching), individual care (training instructions), and physical assessments. Finally, participants were asked to provide open-ended feedback on their impressions of the workshops.

### 2.5. Intervention

Prior to the workshop, a preliminary meeting was held in early December 2023 with four individuals: the department head, a representative from the Human Resources Department, a representative from the General Affairs Department, and the physical therapist responsible for the workshop. During this meeting, several challenges faced by the company were identified, including a high prevalence of chronic pain among employees due to work tasks; a preference for understanding mechanisms due to their professional nature but lack of knowledge about chronic pain and appropriate coping strategies; and a large number of employees with long sitting times and low physical activity. Additionally, because promoting health management aligns with the company’s overall policy, this workshop was intended to be used as a starting point for advancing health management initiatives throughout the company. Based on these issues and aspirations, the intervention plan was designed.

On the day of the workshop, a physical therapist visited the company to conduct the session. It was held in the office’s seminar house over approximately two hours during the morning work hours. The workshop was delivered in a hybrid format, allowing participants to choose between in-person and online participation based on their convenience.

The physical therapist, who normally works at an orthopedic clinic, has experience conducting group workshops for citizens, students, and other physical therapists, but this was their first time conducting a workshop for corporate employees. The content of the workshop was developed based on prior research and proven effective methods [7,8,9,10,23,24].

The content of the workshop was as follows:

1. Impact of chronic pain on labor productivity;
2. Chronic pain retention in the workers;
3. Current treatments of chronic pain in the workers;
4. Simple feedback on the results of the pre-survey;
5. Impact of desk work on chronic pain and measures to prevent it;
6. Biomechanics-based explanation of desk and heavy lifting postures,
  including correct positioning of monitors, head, forearms, and hands, as well
  as proper techniques for standing, sitting, and squatting;
7. Practice examples of stretching and training to improve chronic pain
  designed to be performed during desk work, focusing on the cervical,
  scapular, and pelvic regions.

The workshop initially conducted a 90 min lecture covering topics 1 through 5 in detail. This was followed by a 30 min session at the front of the venue where simple practical skills and demonstrations related to topics 6 and 7 were performed. Additionally, in the post-workshop meeting with the company, an overview of the entire department was presented in a manner that did not identify individuals, and there was a discussion reviewing the workshop as well as future directions.

### 2.6. Data Analysis

The collected data were anonymized and extracted from Google Forms and paper forms. Initially, the average values and standard deviations for presenteeism were calculated based on the presence of chronic pain in at least one body part and specifically for chronic pain in the lower back, shoulders, and neck. Next, literacy scores were determined by summing the correct answers to seven questions, and average scores and standard deviations were calculated based on the presence of pain in at least one body part and by age group. Before analyzing the data, the normality of each variable was assessed. For presenteeism values, all *p*-values were above 0.05 for the lower back and shoulders, suggesting a normal distribution, and differences in means based on the presence of pain were compared using independent *t*-tests. For the neck and cases with pain in at least one body part, some variables did not follow a normal distribution, so the Mann–Whitney U test was used to compare mean differences. For literacy scores, all *p*-values were above 0.05, indicating a normal distribution. Differences due to the presence of pain in at least one body part and among different age groups were compared using independent *t*-tests and one-way ANOVA, respectively. Statistical analyses were conducted using SPSS Statistics 28.0 (IBM Japan), and data were aggregated and analyzed with a significance level set at less than 5%.

### 2.7. Ethical Considerations

This study was conducted in accordance with the guidelines of the Declaration of Helsinki and was approved by the Ethics Committee of Kanazawa Orthopedic and Sports Medicine Clinic (Kanazawa-OSMC-2022-007). At the beginning of the pre-survey questionnaire administered via Google Forms, the purpose of the research, methodology, voluntary participation, and data anonymity were clearly stated. Participants could proceed to answer the questionnaire only after indicating their informed consent. The post-survey also included explanations about the research purpose and data handling, ensuring anonymity. The collected data were anonymized, analyzed, and reported in a manner that did not reveal individual identities. Participants were informed that they could freely choose to participate in the study and could withdraw at any time. Furthermore, it was promised that the results would be used solely for academic purposes.

## 3. Results

As mentioned earlier, the target sample size was 128 participants. However, only 56 individuals completed the pre-survey questionnaire, and the target was not achieved. Table 1 presents the basic attributes of the participants in the preliminary questionnaire survey. Thirty participants completed the post-workshop questionnaire survey.

Table 2 presents the distribution of individuals experiencing chronic pain in at least one body part, broken down by overall prevalence, age group, and specific body parts. Overall, 31 of 56 participants (55.4%) reported experiencing chronic pain. When analyzed by age group, the prevalence was the highest among those aged > 50 years with 11 of 12 (91.7%) affected; followed by those in their forties, with 12 of 22 (54.5%); those in their twenties, with 4 of 9 (44.4%); and those in their thirties with 4 of 13 (30.8%) affected. Examination of the body parts revealed that shoulder pain was the most common affecting 19 of 56 (33.9%) individuals, followed by neck pain affecting 18 of 56 (32.1%), and lower back pain affecting 15 of 56 (26.8%).

Table 3 lists average presenteeism scores based on the presence or absence of pain. The overall presenteeism score was not reported by four participants, and the average was 82.8% (standard deviation = 16.8). Differences in mean values based on the presence or absence of pain in the lower back and shoulders were compared using independent *t*-tests, and no significant differences were found. Additionally, differences in mean values based on the presence or absence of pain in at least one body part and specifically in the neck were compared using the Mann–Whitney U test, and again, no significant differences were observed.

Table 4 presents seven questions related to chronic pain, their correct answers, and the accuracy rates. A tendency for lower accuracy rates was observed in questions dealing with the management of chronic pain. Moreover, scoring was conducted based on the total number of correct answers to the seven questions, resulting in an overall average score of 5.4 points (standard deviation = 1.1). Table 5 lists the average scores differentiated by the presence of chronic pain and by age group. The average score for participants with chronic pain in one or more body parts was 5.5 (standard deviation = 1.0), while the average for those without pain was 5.4 (standard deviation = 1.2). An independent *t*-test comparing these groups showed no significant difference (*p* = 0.34). Furthermore, the average scores by age group were as follows: 5.4 (standard deviation = 1.1) for participants in their twenties, 5.0 (standard deviation = 1.5) for those in their thirties, 5.6 (standard deviation = 0.8) for those in their forties, and 5.6 (standard deviation = 0.9) for those aged 50 and above. A one-way ANOVA conducted to compare the differences among the four age groups found no significant differences (F = 0.934, *p* = 0.431).

Table 6 presents the number and percentage of responses to each question after the workshop (*n* = 30). Table 7 demonstrates the number and percentage of responses to questions regarding the types of services desired by IPT (*n* = 28, with two non-responses). The distribution was as follows: workshops, 14 (50.0%); individual care (massage and stretching), 10 (35.7%); individual care (training guidance), 7 (25.0%); physical measurements, 6 (21.4%); and individual consultations, 5 (17.9%). Table 8 summarizes representative feedback from the workshop. Many positive comments were received regarding understanding the mechanisms of chronic pain and knowledge of posture; however, there were also requests for more detailed information on specific care methods.

## 4. Discussion

In this study, physical therapists conducted workshops as part of an IPT program aimed at preventing and improving chronic pain among workers primarily engaged in desk jobs. Before and after the workshops, questionnaires were administered to employees, and meetings were held with department heads and HR departments to organize issues related to productivity loss due to chronic pain and consider future developments in IPT. Results from the post-workshop questionnaires indicated that more than 70% of the employees experienced an increase in understanding and satisfaction, with positive feedback on the improvement of knowledge about chronic pain mechanisms and posture. However, there were also requests for more interactive communication and a desire to learn more about specific care methods. Several factors contributed to these outcomes. First, the workshops were designed based on an understanding of employees’ professional characteristics and tendencies toward chronic pain, which were gathered through preliminary meetings with department heads and HR departments. Second, the target group’s prevalent experience of chronic pain was considered a contributing factor. Third, as a future challenge, the focus of the study’s initiatives was primarily on primary prevention through general workshops, indicating a potential requirement for more practical hands-on care methods and individualized attention for employees suffering from chronic pain.

First, for workshop implementation, a preliminary meeting was conducted with four individuals: the department head, a representative from the Human Resources Department, a representative from the General Affairs Department, and the physical therapist who would conduct the workshop. Based on the job descriptions gathered during the meeting, posture guidance based on biomechanics was incorporated into the workshop content for sitting postures and lifting heavy objects, referencing prior research [23,24]. Additionally, considering employees’ preferences for understanding mechanisms and evidence-based approaches, the impact of chronic pain on worker productivity was included in the workshop based on evidence from prior research [9,10]. Furthermore, the preliminary survey results were briefly shared during the workshop to communicate the current state of chronic pain in Company A. These efforts likely led to an alignment between what the company and employees sought and the workshop content, resulting in high satisfaction and positive feedback regarding the improved understanding of mechanisms and knowledge. In a survey conducted in the UK, approximately 56% of employees reported not sharing their musculoskeletal chronic pain with their managers [25]. Similarly, in this initiative, it was often the first time that corporate management became aware of employees’ conditions, suggesting that physical therapists could play a significant role in enhancing communication between employees and the company. These outcomes suggest that preliminary meetings are effective for tailoring interventions to a group’s characteristics when physical therapists intervene in corporations.

In the preliminary survey, a low correct answer rate was observed for questions related to knowledge of managing chronic pain, indicating a trend toward an insufficient understanding of chronic pain mechanisms and management among participants. Health literacy encompasses the skills required to access, understand, and utilize necessary information. In this context, we specifically explored knowledge about chronic pain management. Individuals with lower health literacy experienced significantly higher levels of chronic pain [16]. Although the relationship has not been clearly established at this point, the report suggests that low health literacy may contribute to poor pain management knowledge and a lack of coping strategies, potentially affecting the intensity of pain [16]. This suggests that enhancing chronic pain literacy is crucial for reducing the prevalence of chronic pain, which affects more than 50% of employees in Company A. It indicates that not only understanding the causes and mechanisms but also imparting appropriate coping strategies is important. However, it is important to note that the participants in this instance were primarily male engineers engaged in desk work. While the observed trends in this group suggested certain patterns, variations in workplace environments and gender differences could alter the results and trends in health literacy [26,27]. Therefore, caution is needed as different intervention groups may exhibit varying patterns in chronic pain and health literacy.

Second, the results of the preliminary survey on chronic pain indicated that more than half of the employees experienced chronic pain in at least one body part, which led to satisfaction with the workshop and consideration of future efforts in post-meeting discussions with the company. Feedback from the workshop frequently included positive comments from participants with chronic pain, such as ’I am in pain and I learned a lot’. During a post-workshop meeting with the company management, there was a proposal to expand these initiatives based on the survey results across the entire headquarters. This recognition of the high prevalence of chronic pain among employees is seen as a catalyst for determining future strategic approaches. Previous surveys in Japan have reported chronic pain prevalence rates of 22.9% [21] and 15.4% [20]. Internationally, the prevalence of musculoskeletal disorders among individuals who work on computers for more than four days per week has been reported to be 37.9% [28]. Despite differences in symptom duration and pain severity and considering those with mild symptoms lasting more than three months as experiencing chronic pain, it was evident that a significant number of workers in companies primarily composed of desk workers suffer from chronic pain and desire efforts toward its improvement and prevention. It has been reported that incorporating physical activities such as standing and walking every hour during desk work can reduce the incidence of musculoskeletal disorders by 30% [28]. Given the noted prevalence of prolonged desk work and lack of physical activity within the target company, this strategy shows potential as a future preventative measure. Aerobic exercise at 50–60% of maximum heart rate has been shown to alleviate symptoms of chronic pain [29], and core strength training has been reported to mitigate chronic lower back pain [30]. These findings suggest the need to provide appropriate aerobic exercises and core strength training as part of treatment strategies.

When examining chronic pain by the body part, the shoulders, neck, and back were the most commonly affected, in that order, with over 30% suffering from shoulder and neck pain. Previous reports have shown similar distributions of back, shoulder, and neck pain [20], especially among office workers [31].

The average presenteeism score was 82.8%. This study used a single-item measure developed at the University of Tokyo whose average performance level for the Japanese population was reported to be 85% (15% impairment) [22], suggesting that the participants in this survey closely matched the average performance of Japanese workers. No significant differences were found in presenteeism scores based on the presence of chronic pain although trends suggested that chronic pain in the shoulders and neck might affect presenteeism more than back pain. The annual cost loss per person due to presenteeism has been reported to be the highest for shoulder and neck pain, followed by sleep deprivation and lower back pain [7]. A survey of workers from three different occupations found a significant decrease in presenteeism values among those with musculoskeletal chronic pain, particularly in the neck, shoulders, and lower back [12]. According to the report, industries that predominantly involve standing tasks, such as manufacturing and retail, tend to impose greater strain on the lower back and lower limbs, while desk-bound sectors like finance place more burden on the neck and shoulders [12]. In this survey, many participants were engineers primarily doing desk work, which suggests that the strain on the neck and shoulders during computer tasks could lead to musculoskeletal complaints, particularly affecting presenteeism values for the neck and shoulders. Further investigation is necessary as the results can vary based on job content and sample size.

Nevertheless, in the company targeted by this study, of the 56 workers who participated in the preliminary survey, more than half reported chronic pain in at least one body part, and labor losses due to presenteeism were confirmed at approximately 20%. The company subject to this study has been selected for the Ministry of Economy, Trade and Industry’s “Excellent Corporations for Health Management 2023” White 500 list in Japan. This initiative aims to visualize and socially recognize corporations, both large and small, that excel in health management practices [2]. Additionally, proactive attitudes toward employee health and job satisfaction have been observed during meetings with the HR and General Affairs departments. The inclusion of such a well-managed company in this study on the chronic pain issue suggests that the current efforts in industrial physical therapy in Japan may still be insufficient. Consequently, as requested in post-intervention meetings with the company, further efforts are needed to improve chronic pain management and reduce labor loss.

Third, as a future challenge, although this initiative received a certain level of positive evaluation, participants expressed a desire for more interactive engagement and detailed information on specific care methods. From a preventative medicine perspective, this workshop falls under the category of primary prevention or health promotion. Generally, individual interventions are considered effective as a secondary prevention strategy for patients with moderate to severe chronic pain [32], while group workshops have been reported to be effective as a primary prevention measure for those with mild chronic pain [33]. It has been reported that group instruction can lead to improvements in biochemical data and behavioral changes in exercise more effectively than individual instruction [34,35], making the decision to conduct this workshop for a group valuable as an initial intervention. However, for more than half of the employees suffering from chronic pain, a group workshop alone may not have been sufficient, and there may have been a need for secondary and tertiary prevention measures, such as individual physical therapy screening and coordination with medical institutions. Access to a primary care physician has also been reported to be essential for workers’ health [36]. These reports suggest the necessity of enhancing coordination between the intervening physical therapists and the hospitals or primary care physicians they are affiliated with. Of course, enhancing labor productivity is not solely the responsibility of physical therapists. Effective communication with occupational physicians, industrial health nurses, and occupational health managers is also essential. Furthermore, hosting future group workshops that include practical care methods and interactive communication could provide a more comprehensive offering for IPT [3]. The post-workshop survey indicated a high demand for workshops and individual care, suggesting that increasing the sample size and examining the differences based on the presence of chronic pain could enable the provision of IPT suitable for all stages of prevention, ranging from primary to tertiary care.

### Limitations

This case study explores the implementation and preliminary effectiveness of a workshop aimed at preventing and improving chronic pain within a specific department of a corporation. Consequently, the sample size was smaller than the target specified in the power analysis. Additionally, participation was limited to employees from a specific department within the company who volunteered, and the job types were restricted. Therefore, it is recognized that there are limitations to the generalizability of the results. However, the primary aim of this study was not to validate statistical significance on a large scale, but rather to exploratively evaluate the feasibility of interventions within the company, understand the current situation, and assess preliminary effectiveness. The insights gained are considered valuable foundational information for future large-scale research and the development of intervention programs. Additionally, this survey was cross-sectional, highlighting the importance of continuing to monitor trends in chronic pain, presenteeism, and literacy longitudinally. By expanding the data collection from this single-company case study to include other companies and professions, a clearer pathway can be established for delivering effective industrial physical therapy tailored to corporate needs.

## 5. Conclusions

In this study, physical therapists conducted workshops aimed at preventing and improving chronic pain in corporate workers. Surveys and meetings were conducted before and after the workshops to identify and organize the challenges related to productivity loss due to chronic pain faced by the company. A preliminary survey revealed that more than half of employees experienced chronic pain, showed signs of decreased presenteeism, and had low literacy regarding proper coping strategies. The post-survey achieved a satisfaction rate exceeding 70%. From a preventive medicine perspective, this case study demonstrated potential effectiveness, particularly in primary prevention. However, it also suggested the need for future interventions tailored to secondary and tertiary prevention, including individualized care and coordination with medical institutions to provide appropriately staged preventive measures.

## Figures and Tables

**Table 1:** The basic attributes of the participants (*n* = 56).

	*n*	*%*
Men	51	91.1
Women	5	8.9
Age groups		
20–29 years	9	16.1
30–39 years	13	23.2
40–49 years	22	39.3
50+ years	12	21.4
Exercise habits		
More than three times a week	12	21.4
Twice a week	4	7.1
Once a week	14	25.0
Included none	26	46.4
Short exercises		
Available	20	35.7
Not available	36	64.3

Footnote: *n*, number of participants; *%*, percentage of the total number of participants.

**Table 2:** Distribution of participants with chronic pain by the total, age group, and body part.

	*n*	*%*
At least one body part	31/56	55.4
20–29 years	4/9	44.9
30–39 years	4/13	30.8
40–49 years	12/22	54.5
50+ years	11/12	91.7
Lower back pain	15/56	26.8
Shoulder	19/56	33.9
Neck	18/56	32.1

Footnote: *n*, number of participants, with the numerator representing those with chronic pain and the denominator representing the total number of participants; *%*, percentage of the total number of participants.

**Table 3:** Presenteeism scores by chronic pain status, overall, and by specific body part (*n* = 52).

	Available	Not Available	*z*	*p*
	Mean (%) ± SD	Mean (%) ± SD		
At least one body part	80.6 ± 16.3	85.7 ± 17.8	1.846	0.650
Lower back pain	85.5 ± 9.6	81.9 ±18.8		0.113
Shoulder	77.4 ± 19.4	85.7 ± 15.1		0.711
Neck	77.8 ± 19.9	85.3 ± 15.1	1.715	0.086

Footnote: Mean (*%*), average percentage of presenteeism scores; *SD*, standard deviations; *z*, *z*-value; *p*, *p*-value. Missing values were found in four cases. Mann–Whitney U tests were used for ‘At least one body part’ and ‘Neck’. Independent *t*-tests were used for ‘Lower back pain’ and ‘Shoulder’.

**Table 4:** Seven questions related to chronic pain, their correct answers, and the accuracy rates.

No	Questions	Answers	Accuracy Rates (%)
1	The role of poor posture at work	Agree	98.2
2	Importance of resting	Disagree	41.1
3	Irrelevance of productivity decline	Disagree	91.1
4	Influence of physical stiffness	Agree	94.6
5	Connection to mental health issues	Agree	85.7
6	Necessity of actively moving the painful area	Disagree	67.9
7	Effectiveness of cooling the affected area with patches	Disagree	64.3

**Table 5:** The average scores differentiated by the presence of chronic pain and by age group (*n* = 52).

	Mean ± SD	*p*
At least one body part		
Available	5.5 ± 1.0	0.34
Not available	5.4 ± 1.2	
Age groups		
20–29	5.4 ± 1.1	0.43
30–39	5.0 ± 1.5	
40–49	5.6 ± 0.8	
50+	5.6 ± 0.9	

Footnote: Mean, average literacy score; SD, standard deviations; *p*, *p*-value. Missing values were found in four cases. Independent *t*-tests were used for ’At least one body part’, and one-way ANOVA was used for ’Age groups’.

**Table 6:** Each question after the workshop (*n* = 30).

	*n*	*%*
*Q*1. Have you gained new knowledge about chronic pain?		
Strongly agree	23	76.7
Agree	7	23.3
Neutral	0	0.0
Disagree	0	0.0
Strongly disagree	0	0.0
*Q*2. Did you have a good understanding the content?		
Strongly agree	23	76.7
Agree	7	23.3
Neutral	0	0.0
Disagree	0	0.0
Strongly disagree	0	0.0
*Q*3. Do you plan to put into practice what you have content?		
Strongly agree	24	80.0
Agree	5	16.7
Neutral	1	3.3
Disagree	0	0.0
Strongly disagree	0	0.0
*Q*4. How satisfied are you with the workshop?		
Strongly satisfied	21	70.0
satisfied	8	26.7
Neutral	0	0.0
dissatisfied	1	3.3
Strongly dissatisfied	0	0.0

Footnote: *Q*, question; *n*, number of respondents; *%*, percentage of the total number of participants.

**Table 7:** The services desired from industrial physical therapy (*n* = 28).

	*n*	*%*
Workshop	14	50.0
Individual consultations	5	17.9
Individual care (such as massage or stretching)	10	35.7
Individual care (training instructions)	7	25.0
Physical assessments	6	21.4

Footnote: *n*, number of respondents; *%*, percentage of the total number of participants.

**Table 8:** A summary of representative feedback from the workshop.

Now that we understand the mechanisms, we want to start being aware of them.
It was good to learn about stretching and the reasons for pain in an easy-to-understand way.
I am in pain and I learned a lot. Thank you very much.
It was easier to recognize the correct posture after hearing about sciatic and other issues.
It was good because I was able to take in information that I did not know in my desk-bound position.
It would have been better if the classroom sessions were more interactive, such as quizzes and questions.
For example, what exactly should be done to care for the neck? It would have been nice to know a bit more, for example.

## Data Availability

The data presented in this study are available on request from the corresponding author due to privacy and ethical constraints.
